# How body postures affect gaze control in scene viewing under specific task conditions

**DOI:** 10.1007/s00221-023-06771-x

**Published:** 2024-02-01

**Authors:** Daniel Backhaus, Ralf Engbert

**Affiliations:** 1 Department of Psychology, University of Potsdam, Karl-Liebknecht-Str. 24-25, Potsdam, 14476 Germany; 2 Research Focus Cognitive Sciences, University of Potsdam, Karl-Liebknecht-Str. 24-25, Potsdam, 14476 Germany

**Keywords:** Natural scene viewing, Mobile eye-tracking, Postural variation, Task variation

## Abstract

Gaze movements during visual exploration of natural scenes are typically investigated with the static picture viewing paradigm in the laboratory. While this paradigm is attractive for its highly controlled conditions, limitations in the generalizability of the resulting findings to more natural viewing behavior have been raised frequently. Here, we address the combined influences of body posture and viewing task on gaze behavior with the static picture viewing paradigm under free viewing as a baseline condition. We recorded gaze data using mobile eye tracking during postural manipulations in scene viewing. Specifically, in Experiment 1, we compared gaze behavior during head-supported sitting and quiet standing under two task conditions. We found that task affects temporal and spatial gaze parameters, while posture produces no effects on temporal and small effects on spatial parameters. In Experiment 2, we further investigated body posture by introducing four conditions (sitting with chin rest, head-free sitting, quiet standing, standing on an unstable platform). Again, we found no effects on temporal and small effects on spatial gaze parameters. In our experiments, gaze behavior is largely unaffected by body posture, while task conditions readily produce effects. We conclude that results from static picture viewing may allow predictions of gaze statistics under more natural viewing conditions, however, viewing tasks should be chosen carefully because of their potential effects on gaze characteristics.

**Supplementary Information:**

The online version contains supplementary material available at 10.1007/s00221-023-06771-x.

## Introduction

The area of high-acuity vision, the fovea centralis, corresponds to about $2^\circ$ of visual angle. As a consequence, we have to change our gaze frequently to process detailed visual information from the environment. In everyday tasks, human observers perform combined eye, head, and trunk movements for gaze shifts (Land et al. 1999) to keep eye movements within a comfortable range of up to about $25^\circ$ (Stahl 1999), while the maximum range of saccade amplitudes is approximately $\pm 55^\circ$ (Guitton and Volle 1987). However, even for smaller movements ($<15^\circ$), we produce coordinated eye and head movements under natural conditions (Franchak et al. 2021; ’t Hart and Einhäuser 2012). The physiological basis for coordinated eye, head, and postural movements is given by the neural coding of gaze positions (Paré et al. 1994). The proportion to which a subject uses eye or head movements for gaze shifts, however, varies greatly between individuals (Pelz et al. 2001).

The fact that coordination of eye, head, and trunk is ubiquitous in everyday situations, is in contrast with the static picture viewing paradigm, the well-established method to study visual scene exploration through gaze shifts. In the static picture viewing paradigm, gaze behavior on real-world scenes is investigated in darkened laboratory setups (Henderson 2003; Rayner 2009), where a stationary eye tracker is employed, participants are seated, typically with a head-supporting chin rest, and gaze shifts are produced by eye movements only and are practically restricted to the limits of the computer screen size.

While the static picture viewing paradigm has yielded many insightful results, the restrictions have always been criticized (e.g., Tatler et al. 2011), in particular, with the arising new technological progress to obtain high-resolution eye-tracking data in real-world situations. For a good overview of the critique, see Tatler et al. (2011) and Henderson (2003, 2006, 2007). The papers criticizing the static picture viewing paradigm not only question the generalizability of laboratory studies to real-world behavior, but also discuss the frequent lack of a concrete task, the sudden onset of the scene, the relatively short viewing time, the limited field of view, the lack of depth and motion cues, the limited dynamic range, and the photographer bias. A growing literature is investigating aspects between the laboratory and the real-world (e.g., Foulsham et al. 2011; Gert et al. 2022).

Well-established effects on gaze statistics were discovered with the static picture viewing paradigm. A prominent example is the central fixation bias (Tatler 2007; ’t Hart et al. 2009), which is strongest for sudden image onsets (Rothkegel et al. 2017). The participant’s gaze is biased toward the center of a given image, particularly at the beginning of scene exploration, i.e., for the first few saccades. But also later during the trial, fixations at central locations are disproportionately frequent, independent of the positioning of the image on the monitor (Bindemann 2010) and independent of the distribution of salient locations on the image. Even the starting position (central vs. non-central) has little influence to reduce the central fixation bias as long as sudden image onsets were applied (Tatler 2007; Rothkegel et al. 2017). Rothkegel et al. (2017) were able to reduce the strength of the central fixation bias by introducing a short preview time to the scene.

The interactions of eye, head, and trunk movements have been studied extensively (e.g., Stahl 1999; Imai et al. 2001; Pelz et al. 2001; Land 2004; Franchak et al. 2021). Among the key questions is the problem whether the addition of head and body movements is merely compensatory or whether the gaze positions and fixation times are modulated, when observers are permitted to produce head and body movements. Results are inconclusive. For example, Smith et al. (2019) found shorter search times in a visual search task in standing than in sitting, but this result was obtained without eye tracking and the effect occurred in the easier of two search conditions only. Other results of body posture manipulation on cognitive components produced ambiguous results. For example, the color stroop effect (Stroop 1935) was reduced in some studies (Rosenbaum et al. 2017, 2018; Smith et al. 2019; Caron et al. 2020). However, a meta-analysis and a replication showed that these findings cannot be confirmed (Straub et al. 2022).

In addition to postural influences, gaze movements are also dependent on the viewing task (Schwetlick et al. 2023). Early anecdotal findings date back to Buswell and Yarbus who found first differences in the gaze movements during picture viewing when viewing instructions of the observers were varied (Yarbus 1967; Buswell 1935). More recent research investigated the influence of instruction under controlled experimental procedures (e.g., Backhaus et al. 2020; Castelhano et al. 2009; Torralba et al. 2006). Additionally, knowledge of scenes and targets were analyzed (Mills et al. 2011; Kaspar and König 2011; Trukenbrod et al. 2019) with respect to their influence on gaze control.

The present work aims to investigate the generalizability of results from static picture viewing paradigm to less restricted posture under different tasks. In Experiment 1, a within-subject design is applied to analyze viewing behavior during sitting and standing in a free viewing and a more specific viewing task. In Experiment 2, we investigate viewing behavior under four different postural manipulations, from highly restricted to more flexible postures. Effects on gaze behavior are analyzed separately for temporal and spatial viewing characteristics. Since we are interested in the question of whether there are any differences between conditions, we do not formulate directed hypotheses.

## Experiment 1

In the first of our experiments, we investigate the influence of two different body postures on gaze behavior under two different task conditions. The static picture viewing paradigm is typically investigated during sitting with chin rest support (*Chin_Rest*) without a concrete viewing task (*Free_Viewing*). We contrast this setup with the postural condition of quiet standing (*Standing*) and a more specific task condition, where participants were required to guess the time of the day the image was taken (*Guess_Time*), a task we used with the same image material in an earlier study (Backhaus et al. 2020). We chose this task since it is a slightly more concrete task than free viewing (subjects can develop their own strategy for extracting time from the picture) and there is no clear presumption of attentional locations in the picture. As a result, we apply a $2\times 2$ within-subject design.

### Methods

#### Participants

Thirty-one students (26 females, 5 males, age range from 19 to 49 years, mean age $=$ 25.2 years) with normal or corrected-to-normal vision participated in this experiment. An additional eight students were excluded from the analyses since the experiment had to be stopped during the recording because of persistent calibration failures ($n=5$) or reported uneasiness ($n=2$). Another participant ($n=1$) was excluded due to abnormal fixation patterns produced during the experiment. Participants were recruited via a departmental internal portal and received credit points or monetary compensation (€ 9.00). To increase engagement with the task, we offered participants an additional incentive of up to € 1.50 for correctly answering questions after 30 of the 60 images. The study was carried out in accordance with the Declaration of Helsinki. Written informed consent was obtained for experimentation by all participants prior to testing.

#### Apparatus and saccade detection

Stimulus images were presented on a luminance-calibrated projector (JVC DLA-X9500B; Victor Company of Japan Ltd., Yokohama, Japan) with a refresh rate of 60 Hz and a resolution of 1920$\times$1080 pixels. Participants were placed at a distance of 270 cm from the projector screen in all experimental conditions, i.e., during sitting and standing. Infrared video-based mobile eye-tracking glasses (SMI-ETG 2W, SensoMotoric Instruments, Teltow, Germany) were used to record participants’ eye movements during the experiment. Gaze positions were obtained binocularly in scene camera coordinates on a sub-pixel level with a sampling rate of 120 Hz. Scene camera resolution was 960$\times$720 pixels (or $60^\circ \times 46^\circ$ visual angle) with a refresh rate of 30 Hz. Figure S1 in the supplement shows the experimental setup in our laboratory. For saccade detection, we transformed data from scene camera coordinates to stimulus image coordinates (cf., Backhaus et al. 2020). Next, we used both binocular gaze trajectories and applied a velocity-based algorithm (Engbert and Kliegl 2003; Engbert and Mergenthaler 2006) with the same set of parameters as reported in Backhaus et al. (2020). Gaze position was computed using the binocular stream provided by the hardware. After saccade detection, fixations were defined as time intervals between subsequent saccades. Saccade metrics were defined from gaze shifts on stimulus images irrespective of the differentiation between eye-in-head and head-in-space movements. The eye tracker detection was used to label the blinks. Both blinks and the preceding and succeeding events (i.e., fixations or saccades) were excluded from further analysis.

#### Materials and procedure

Natural photographs with a resolution of 1668$\times$828 pixels were presented in the center of the screen. Spatial extent of the stimulus images covered $40.6^\circ$ of visual angle in the horizontal and $20.1^\circ$ in the vertical dimension. For later screen detection, stimulus images were embedded in a grey frame that included 12 unique QR-markers (126$\times$126 pixels each). Colored photographs were taken from Backhaus et al. (2020). The photographs contained varying numbers of humans and animals (between 0 and 10), having overall sharpness, no prominent text, and are taken in different countries and on different daytime.

**Fig. 1:**
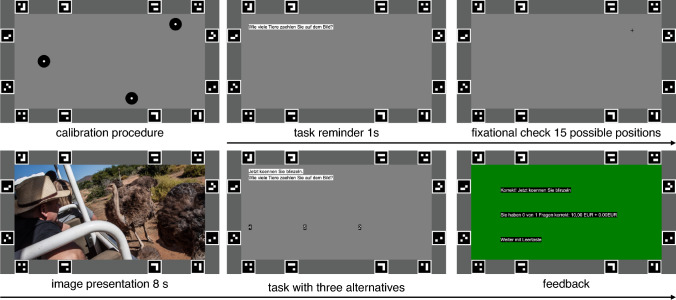
Experimental paradigm for Experiment 1 and 2: Schematic sequence of an experimental trial. The black arrow represents the temporal sequence. Task-relevant displays are omitted during free viewing conditions

Experiment 1 consisted of four Blocks of fifteen images with a presentation time of 8 s each. In the first two Blocks, participants viewed 30 images in randomized order under task condition *Free_Viewing*, where subjects did not have a specific task instruction. The second manipulated factor was body posture with the variations of sitting with a chin rest (*Chin_Rest*) versus standing quietly (*Standing*). Note that screen height was adjusted to participants’ vertical eye positions in space. Body posture conditions were counterbalanced and assigned to Block A and Block B. In Block C and Block D participants viewed the 30 images for a second time in randomized order, but under the specific task condition of asking the subject to guess the time of the day the image was taken (*Guess_Time*). Body posture conditions were again counterbalanced and assigned to Block C and Block D. Every session started with detailed instructions of the upcoming task followed by a calibration. Trials consisted of a screen with a task reminder (1 s), followed by a fixation check (3 s), and the image presentation (8 s). In Blocks C and D, three alternative answers to the guessing task were presented. Participants were required to answer verbally (condition *Standing*) or by knocking on the table while fixating on the selected answer with their eyes (condition *Chin_Rest*). We have chosen this modality because speaking is not possible while the head and chin are fixed. The experimenter entered the answers into the computer. Correctly answered questions were rewarded with an incentive of € 0.05. The specific query and the reward serve to maintain the motivation of the participants. Therefore, we did not analyze the actual answers. Participants guessed the time correctly in 65 % of the guessing trials. A schematic sequence of an experimental trial is shown in Fig. 1.

Throughout the experiment, participants’ eye movements were recorded. For calibration, we used the SMI built-in 3-point calibration routine after at least every fifth trial or whenever the experimenter decided to recalibrate. For fixation checks, a black cross ($0.73^\circ \times 0.73^\circ$) on medium gray background appeared on a randomly selected position (from 15 possible positions defined by three vertical positions between 25% and 75% of the projector screen’s vertical size and five horizontal positions between 20% to 80% of the projector screen’s horizontal size). The experimenter started a calibration, whenever the eye position deviated more than about $1^\circ$ of visual angle from the initial fixation target at the beginning of each trial.

#### Data preprocessing

Since mobile eye-tracking signals are typically noisier than signals recorded via desktop devices, we applied a list of exclusion criteria to remove unreliable events during preprocessing. Blinks detected by the eye tracker, fixations shorter than 33 ms (equivalent to four samples), fixations with durations greater than or equal to 1000 ms and fixations with jittering signals (which exceeded the 2D median standard deviation of all fixations by a factor of 15) as well as saccades with amplitudes greater than $25^\circ$ were detected. In all these cases, we removed the events as well as the neighboring events before and after the critical event. Finally, all trials where gaze positions deviated greater than $2^\circ$ from the fixation target during the last 200 ms before image presentation were discarded.

#### Statistical analyses

For our statistical analysis, our approach is based on linear mixed models. This method allows us to include experimentally varied factors (fixed effects), covariates, as well as within-design groups (random effects) in one model. Our orthogonal contrasts of the fixed factors reflect our hypotheses about the varied body postures and tasks. As within-grouping factors, we integrate (whenever possible) the subjects and the presented images. The complexity of these random factors is chosen according to the recommendations of Bates et al. (2018) and Matuschek et al. (2017). For some dependent variables, a simpler random effect structure with only the intercept for the subjects and the intercept for the images is chosen for better comparability between models. The analysis was performed with R (v.4.2.1, R Core Team 2022) and the lme4 package (v.1.1-30, Bates et al. 2015). Models were estimated using maximum likelihood and the Bobyqa optimizer; *p*-values were calculated with the lmerTest package (v.3.1-3, Kuznetsova et al. 2017). In the presentation of results, we focus on the experimentally varied fixed effects, which are controlled for between-subject and between-image variances through the random effects. Details about the random effect variances can be found in the provided code. The resulting models for each dependent variable can be found in Table 1.

**Table 1:** Linear mixed-effects model structure

Dependent variable	Fixed effect part	Random effect part	
*Experiment 1*			
Log(Fixation Duration) $\sim$	1 + Task + Body + Task:Body	+ (1 | Subj)	+ (1 | Img)
Log(Saccade Amplitude) $\sim$	1 + Task + Body + Task:Body	+ (1 | Subj)	+ (1 | Img)
Exp(Entropy) $\sim$	1 + Task + Body + Task:Body		+ (1 + Task || Img)
Distance to Image Center $\sim$	1 + Task + Body + Task:Body + scale(fixcrossDeg, center = StartDistanceMean) + scale(log(sample))	+ (1 | Subj)	+ (1 | Img)
*Experiment 2*			
Log(Fixation Duration) $\sim$	1 + C1 + C2 + C3	+ (1 | Subj)	+ (1 | Img)
Log(Saccade Amplitude) $\sim$	1 + C1 + C2 + C3	+ (1 | Subj)	+ (1 | Img)
Exp(Entropy) $\sim$	1 + C1 + C2 + C3		+ (1 + C3 || Img)
Distance to Image Center $\sim$	1 + C1 + C2 + C3 + scale(fixcrossDeg, center = StartDistanceMean) + scale(log(sample))	+ (1 | Subj)	+ (1 | Img)

Note: The Wilkinson notation of the model equations are as follows: “1” represents the intercept which is the grand mean in the fixed effect part and the subjects’ and images’ means in the random effect part. “Task” is the contrast that captures the difference between the two task conditions ${ Guess\_Time} - { Free\_Viewing}$, “Body” is the contrast that captures the difference between the two body posture conditions ${ Standing} - { Chin\_Rest}$, and “Task:Body” is the interaction of the factors Task and Body. “C1” is the contrast that captures the difference between sitting postures versus standing postures $({ Standing}~\&~{ Balancing}) - ({ Chin\_Rest}~\&~{ Sitting})$, “C2” is the contrast that captures the difference within sitting postures $({ Sitting}) - ({ Chin\_Rest})$, “C3” is the contrast that captures the difference within standing postures $({ Balancing}) - ({ Standing})$. The term “scale(fixcrossDeg, center = StartDistanceMean)” is a covariate that controls for the starting position of each trial, “scale(log(sample))” is a covariate that controls for the logarithmic trend of the dependent variable over time. “Subj” is the grouping variable for the subjects, “Img” is the grouping variable for the images, “||” is a parameter that suppresses the calculation of correlations between random effect model parameters

### Results

We investigated effects of body posture and task on different gaze parameters. First, we report effects for temporal parameters (fixations durations), and, second, we report obtained effects on spatial parameters (saccade amplitudes, gaze distribution via entropy, central fixation bias).

#### Temporal parameters

**Fig. 2:**
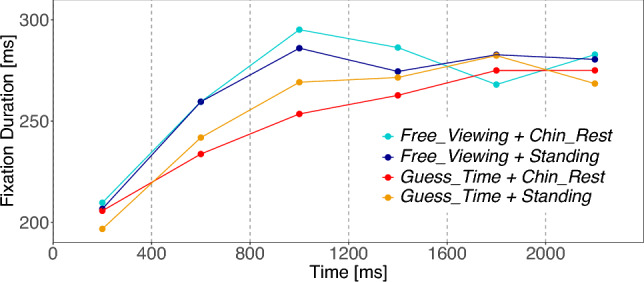
Experiment 1: Mean fixation duration for the first 2 s of image presentation. Each line corresponds to one of the four conditions. Means are calculated in bins of 400 ms

We analyzed the fixation duration, i.e., the key temporal parameter of gaze behavior, across the different experimental conditions (see Table 2). In a linear mixed effect analysis, we considered (A) the difference between the two task conditions (*Task*), (B) the difference between the two body posture conditions (*Body*), and (C) the interaction of predictors A and B (*Interaction*) as fixed effects. In addition, we controlled for the influence of the subjects and images by including one intercept estimate for both subject and image in the model as varying (random) components (see Methods, Table 1). We limited our analysis to the first 2 s of image presentation, since later effects were not expected (Table 2). Furthermore, the first fixations (on the fixation cross) were excluded from our analyses. Figure 2 visualizes the mean fixation durations over the initial 2 s of image viewing time. Table S1 in the supplement shows the results of the linear mixed effect model (LMM) analysis. Fixation durations were log transformed to better conform to the normal distribution assumptions of the residuals. Note, that *t*-values above 2 are considered significant results. We found a significant effect for the task contrast with longer fixation durations for the free viewing task [*Task*: $M=-0.03$; $SE=0.01$; $t=-3.34$]. No other contrast reaches significance level.

**Table 2:** Experiment 1: Descriptive means

	Experiment 1			
	Chin_Rest	Standing	Guess_Time	Free_Viewing
Fixation Duration 0–2000 (ms)	255.532	257.098	249.926	263.107
Fixation Duration 0–8000 (ms)	269.224	268.150	268.860	268.511
Saccade Amplitude 0–2000 ms ($\phantom{}^\circ$)	6.21362	6.24796	6.32367	6.13299
Saccade Amplitude 0–8000 ms ($\phantom{}^\circ$)	6.13067	6.18681	6.27037	6.04584
Entropy (bit)	13.3745	13.3235	13.3107	13.3873
CFB 0–8000 ms ($\phantom{}^\circ$)	9.36070	9.10227	8.97923	9.48900
CFB 0–400 ms ($\phantom{}^\circ$)	6.38302	6.34617	6.33002	6.40243
CFB 400–800 ms ($\phantom{}^\circ$)	6.93425	6.79592	6.65128	7.06954
CFB 800–1200 ms ($\phantom{}^\circ$)	8.06207	8.29271	8.25300	8.09131
CFB 1200–8000 ms ($\phantom{}^\circ$)	9.74897	9.45199	9.31812	9.88802

Note: Mean values of the dependent variables in their original matrices for the four experimental conditions of Experiment 1

#### Spatial parameters

For the evaluation of spatial gaze characteristics, we examined the following metrics: saccade amplitudes, entropy of the fixation location density, and mean distance to the image center. For all analyses, we calculated linear mixed models (LMMs) with the same fixed effect structure as for the temporal parameters (see Methods, Table 1). The LMM variance components differ for entropy. Since entropy can only be calculated per image, subject variance components are excluded. However, we have added a slope estimation for the task contrast next to the intercept in the image variance components part, which resulted from the model selection procedure (Bates et al. 2018). For the distance to the image center analysis, we added two covariates which significantly improved the log-likelihood of the LMMs.

**Fig. 3:**
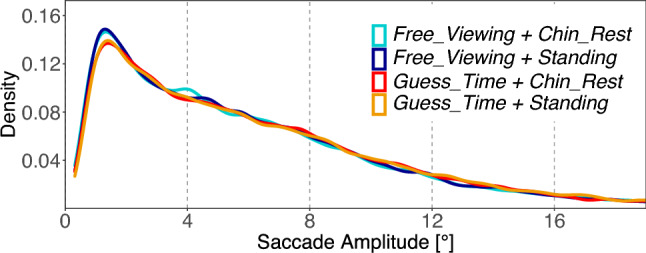
Experiment 1: Distribution of saccade amplitudes. The figure shows relative frequencies of saccade amplitudes in the four conditions. Density is estimated with Gaussian smoothing kernel, bandwidth is defined as half of the standard deviation

Saccade amplitudes were log transformed to better conform to the normal distribution assumptions of the residuals. Over the total viewing time of 8 s, we find a significant difference in log saccade amplitudes for the *Task* contrast (Fig. 3). The free viewing task induces shorter log amplitudes than the guessing task [*Task*: $M=0.05$; $SE=0.01$; $t=5.18$]. No other contrast reached the significance level. Looking only at the first 2 s of image viewing, the same pattern emerged (see Table S2 in the supplement).

The entropy is an information measure (Shannon and Weaver 1963) of the distribution of fixation locations on an image. First, we transformed the fixation location density into a probability $p_i$ of a grid (128$\times$128 cells) with $\sum _i p_i=1$. The entropy is computed as

1 $$\begin{aligned} {S} = - \sum \limits _{i=1}^{n}p_i \log _2 p_i. \end{aligned}$$

Thus, the entropy is measured in bits and ranges from 0 to $\log _2(128^2)=14$ bits, where the maximum corresponds to an equal distribution over the cells. Finally, we exponentially transposed the entropy values to better conform to the normal distribution assumptions of the residuals.

**Fig. 4:**
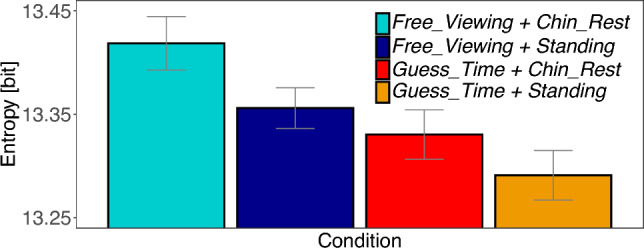
Experiment 1: Shannon’s entropy. Average entropy of fixation densities on an image in the four conditions. A value of 14 bit is expected for a uniform fixation density. Smaller values indicate that fixations cluster in specific parts of an image. Confidence intervals were corrected for within-subject designs (Cousineau 2005; Morey 2008)

We find significant differences between tasks [*Task* :  $M=-43129.69$; $SE=15391.02$; $t=-2.80$]. Free viewing task produces a larger entropy and thus a wider distribution of fixation locations across the image. Furthermore, we found a significant influence of body posture [*Body* :  $M=-31005.46$; $SE=10550.38$; $t=-2.94$]. When sitting with chin rest, the subjects spread their gaze further over the image compared to the standing position (see Table S3 in the supplement). No interaction was found [*Interaction* :  $M=7607.86$; $SE=21100.76$; $t=0.36$]. Figure 4 shows the differences in the original metric.

The distance to the center of the image is a measure for the central fixation bias (Rothkegel et al. 2017; Tatler 2007). To control the influence of the starting position (fixation cross), we sampled the data in such a way that all 15 starting positions were present equally often in all four conditions. We excluded the fixation on the fixation cross from the analysis. To control for its influence, we included the distance of the starting position from the image center as a covariate in the LMM. We also added the logarithmized sample number to the model as a further covariate to linearize the change of the CFB over time.

**Fig. 5:**
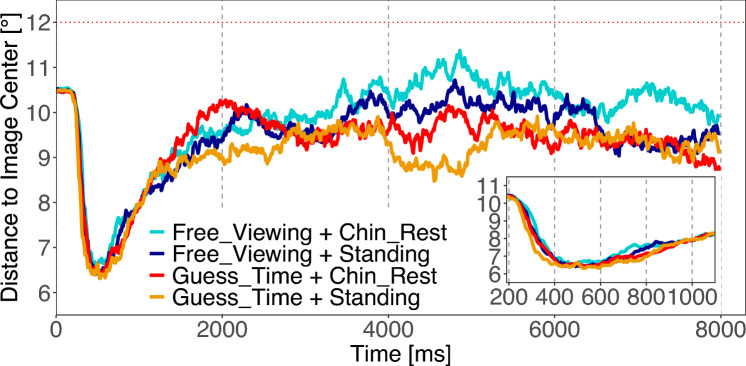
Experiment 1: Temporal evolution of the central fixation bias measured as the average distance to the image center. Each line corresponds to one of the four conditions. $12~^\circ$ represents the expected distance to the image center if fixations were uniformly placed on an image (dotted red line). The inset shows a magnification of the time interval from 200 ms to 1000 ms

Over the whole viewing time, we find a significant difference caused by the different tasks [*Task* :  $M=-0.43^\circ$; $SE=0.05^\circ$; $t=-8.65$]. Free viewing produces a less pronounced bias toward the center of the image. We also find an influence of body posture, while an interaction of task and body posture is absent [*Body* :  $M=-0.28^\circ$; $SE=0.05^\circ$; $t=-5.51$; *Interaction* :  $M=-0.01^\circ$; $SE=0.10^\circ$; $t=-0.13$]. Standing posture produces a stronger bias toward the center of the image. We also examined the evolution of central fixation bias in fine-scaled steps of 400 ms for the early phase to 1200 ms and a separate analysis of the later bias at the viewing time from 1200 to 8000 ms (Fig. 5).

For the later phase (1200 ms to 8000 ms), we find the same effects even more pronounced as for the whole viewing time. In the early viewing phase (0 ms to 1200 ms), we find no influence of the viewing task nor of the body posture, except for the time interval from 400 ms to 800 ms in which the effect of the viewing task already shows up in the same direction as in the later viewing phase. Note that for all analysis the residuals are not normally distributed because of a floor effect. The results can be found in Table S4 in the supplement.

## Experiment 2

In the second experiment, we aim at a more detailed investigation of the possible influence of body postures. Here, we used the same setup as in Experiment 1, but varied the posture over four levels, ranging from strongly restricted sitting with a chin rest support over more natural tasks of normal sitting and normal standing to body postures resulting from standing on a balance board. For the viewing task, we gave a specific task, where participants were required to count the animals in a given image, across all postural conditions. This specific task clearly requires active gaze behavior, it minimizes the variation due to the level of understanding of the task between subjects, and yet does not strongly restrict the fixation locations, as animals can appear all over the image. We applied this task in an earlier study (Backhaus et al. 2020). Note that there was not a variation of task. The selected task serves to ensure that the participants do not choose their own task, thus minimizing task-related variability between subjects.

### Methods

#### Participants

Thirty-two students with normal or corrected to normal vision recruited through an internal university platform participated in this experiment (24 female, 8 male, age range from 18 to 35 years, mean age $=$ 22.7 years). Participants received credit points or monetary compensation (€ 10.00). To increase compliance with the task, we offered participants an additional incentive of up to € 3.00 for correctly answering questions presented after each image. The work was carried out in accordance with the Declaration of Helsinki. Informed consent was obtained for experimentation by all participants.

#### Materials and procedure

The same 30 natural photographs were used as in Experiment 1, with an additional 30 images that fulfilled the same selection criteria. Experiment 2 consisted of four Blocks of fifteen images with a presentation time of 8 s each. In each Block, participants viewed the pictures under the same task condition (*Count_Animals*). Each picture contained 0 to 10 animals. The manipulated factor of Experiment 2 was body posture with four variations: sitting with chin rest support (*Chin_Rest*), sitting without chin rest support (*Sitting*), quiet standing (*Standing*), and standing on a balance board (*Balancing*). Our balancing board consists of two round plastic discs with a diameter of 36 cm. The screen height was adjusted to the eye height of the participants in each Block. Posture conditions were balanced and assigned to Blocks A, B, C, and D between participants. The 60 images were presented in a randomized order. The instruction and calibration procedure were analogous to the procedure in Experiment 1. After each image, participants were presented with 3 alternatives to choose from. Subjects responded verbally in all Blocks except for the Block with the seated chin rest posture, where subjects tapped the table while gazing at their selected answer. We have chosen the verbal response modality to consistently implement a more natural setup. Correctly answered questions were rewarded with an incentive of € 0.05. As in Experiment 1, we do not analyze the participants’ answers. The specific query and the reward serve to maintain the participants’ motivation. Participants responded with the correct number of animals in the picture in 83 % of the trials.

### Results

#### Temporal parameters

**Fig. 6:**
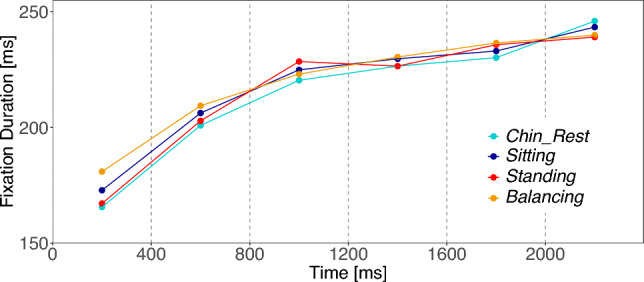
Experiment 2: Mean fixation duration for the first 2 s of image presentation. Each line corresponds to one of the four posture conditions. Means are calculated in bins of 400 ms

We investigated the time course of mean fixation durations across postural conditions (Fig. 6). We examined effects using a linear mixed effects model applying the following three contrasts for the models fixed effect part: (C1) the difference between sitting postures (*Chin_Rest* & *Sitting*) versus standing postures (*Standing* & *Balancing*), (C2) the difference within sitting postures (*Chin_Rest*) versus (*Sitting*), and (C3) the difference within standing postures (*Standing*) versus (*Balancing*). We controlled for the influence of the subjects and images and included one intercept estimate each in the model as variance components (see Methods, Table 1). As in Experiment 1, the analysis is limited to the first 2 s of image viewing. Furthermore, the first fixations on the fixation cross were excluded from the analysis. Table S5 in the supplement shows the results of the LMM analysis. Note, that *t*-values above 2 are considered significant results. None of the three contrasts reaches significance level, thus we find no significant influence of body posture variation on fixation durations (Table 3).

**Table 3:** Experiment 2: Descriptive means

	Experiment 2			
	SittingChinrest	Sitting	Standing	StandingBalance
Fixation Duration 0–2000 ms (ms)	209.474	212.076	211.773	215.757
Fixation Duration 0–8000 (ms)	224.708	223.358	223.585	225.846
Saccade Amplitude 0–2000 ms ($\phantom{}^\circ$)	6.23333	6.30860	6.65130	6.48988
Saccade Amplitude 0–8000 ms ($\phantom{}^\circ$)	6.25075	6.23667	6.40981	6.46980
Entropy (bit)	13.4943	13.4638	13.4773	13.4371
CFB 0–8000 ms ($\phantom{}^\circ$)	10.4669	10.4231	10.4946	10.1095
CFB 0–400 ms ($\phantom{}^\circ$)	6.09431	5.84103	5.85303	5.70179
CFB 400–800 ms ($\phantom{}^\circ$)	7.35974	7.49000	7.56199	7.33750
CFB 800–1200 ms ($\phantom{}^\circ$)	9.37699	9.71689	9.94345	9.07237
CFB 1200–8000 ms ($\phantom{}^\circ$)	10.9451	10.8678	10.9394	10.5717

Note: Mean values of the dependent variables in their original matrices for the four posture conditions of Experiment 2

#### Spatial parameters

As in Experiment 1, we focused on saccade amplitudes, entropy of the fixation location distribution, and distance of fixations to the image center for the analysis of spatial gaze statistics. We applied the same exclusion criteria as in Experiment 1. The fixed effect structure of all LMM analyses was kept the same from the temporal analysis of Experiment 2. For the varying (random) effect structure the entropy model, as before, could not account for subject variance because of its calculation via averaging over subjects. We added the slope for the contrast which captures the difference within standing postures (C3), next to the intercept in the image variance components part according to the model selection procedure of Bates et al. (2018). For the distance to image center analysis, we again added two covariates, which significantly improved the log-likelihood of the models.

**Fig. 7:**
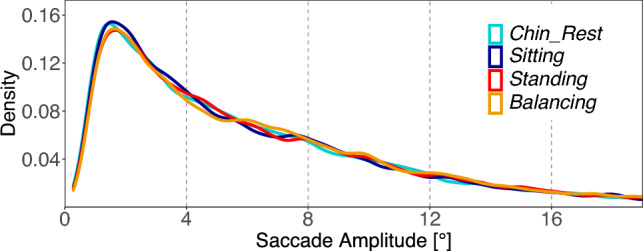
Experiment 2: Distribution of saccade amplitudes. The figure shows relative frequencies of saccade amplitudes in the four posture conditions. Density is estimated with gaussian smoothing kernel, bandwidth is defined as half of the standard deviation

Over the total viewing time of 8 s, we find a significant difference in log saccade amplitudes in the first contrast (C1) which captures the difference between sitting postures and standing postures. While participants were sitting (independent of chin rest support), smaller saccade amplitudes are generated compared to the standing postures (Fig. 7) [C1 $standing\ postures - sitting\ postures$: $M=0.03$; $SE=0.01$; $t=4.09$]. No other contrast reached the significance level. An analysis limited to the first 2 s of viewing time generated the same pattern of results (cf. Table S6 in the supplement).

**Fig. 8:**
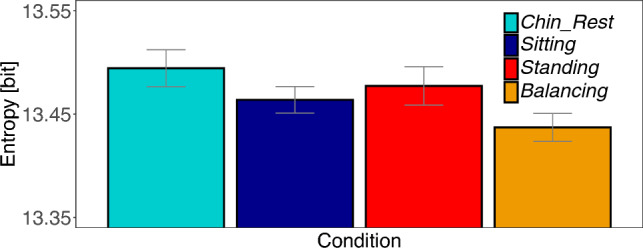
Experiment 2: Shannon’s entropy. Average entropy of fixation densities on an image in the four posture conditions. A value of 14 bit is expected for a uniform fixation density. Smaller values indicate that fixations cluster in specific parts of an image. Confidence intervals were corrected for within-subject designs (Cousineau 2005; Morey 2008)

For entropy, we applied an exponential transformation of the numerical values to achieve a closer fit to the assumption of normal distributed residuals. We found a trend that sitting conditions produced larger entropy values, thus fixation locations are more uniformly distributed over the image compared to the standing conditions. This difference, however, is not statistically reliable as the *t*-value did not reach the significance level [C1 $standing\ postures - sitting\ postures$: $M=-10101.68$; $SE=8252.99$; $t=-1.22$]. The other two contrasts reveal significant differences, but these should also be treated with caution, as the *t*-values are narrowly above the significance level. Sitting with a chin rest produced larger entropy values in comparison with head-unrestrained sitting [C2 $Sitting - Chin\_Rest:$ $M=-23962.39$; $SE=11671.48$; $t=-2.05$]. Quiet standing produced larger entropy values compared to standing on a balance board [C3 $Balancing-Standing$: $M=-31856.52$; $SE=13684.64$; $t=-2.33$] (cf. Table S7). The differences in the original metric are shown in Fig. 8.

For the analysis of the central fixation bias, we used a LMM model structure that can be applied to all different time intervals. Again, we chose a minimal varying (random) structure with only subject intercept and image intercept (see Table 1). For the early time intervals, there is little subject variance. For later time intervals (1200 ms to 8000 ms), the models with subject-varying effects explain the data better but do not differ from the simpler models in direction and significance at the level of $\alpha =0.05$. As in Experiment 1, we sampled the data in such a way that all 15 starting positions were present equally often in all four conditions and excluded the fixations on the fixational cross itself. We included both covariates that we used in Experiment 1 to control for the distance of the starting position from the image center as well as we linearized the change of the CFB over time by using logarithmic sample number as a covariate.

**Fig. 9:**
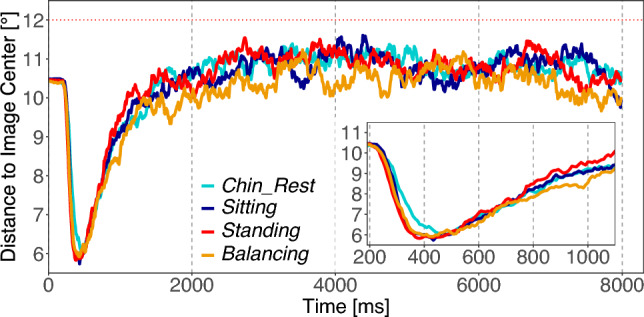
Experiment 2: Temporal evolution of the central fixation bias measured as the average distance to the image center. Each line corresponds to one of the four posture conditions. $12~^\circ$ represents the expected distance to the image center if fixations were uniformly placed on an image (dotted red line). The inset shows a magnification of the time interval from 200 ms to 1000 ms

Over the entire viewing time of 8 s, we find significant differences in two contrasts due to the more centrally placed fixations elicited by the standing on a balance board condition [C1 $standing\ postures - sitting\ postures$: $M=-0.15$; $SE=0.05$; $t=-2.89$; C2 $Balancing-Standing$: $M=-0.26$; $SE=0.07$; $t=-3.52$](Fig. 9). We also examined the evolution of the central fixation bias in fine-scaled steps of 400 ms, for the early phase up to 1200 ms, and conducted a separate analysis of the later bias during the viewing time from 1200 ms to 8000 ms. Therefore, we conclude that the differences mainly occur in the later phase of image exploration. Additionally, in a very early viewing phase (0 ms to 400 ms), we found a significant difference between the sitting conditions and the standing conditions (C1) in the same direction, with standing conditions producing more centrally placed fixation locations than sitting conditions. Note, that for all analyses the residuals are not normally distributed because of a floor effect. The results can be found in Table S8 in the supplement.

## Discussion

In the current study, we investigated the influence of postural manipulations on gaze behaviors for natural scene perception under different viewing tasks. Postural manipulations were sitting with head-support by a chin rest, head-unrestrained sitting, quiet standing, and standing on a balance board. Viewing tasks investigated were free viewing (i.e., no instruction), a guessing task (guessing the time of the day the image was taken), and a counting task (count the number of animals shown in the image). Different postures did not produce significant differences in fixation durations (temporal parameters). We found, however, that body postures had an impact on spatial parameters. Less controlled postures (such as standing) led to gaze positions that were more clustered at specific locations, and were closer to the image center, in particular, for the later viewing phase ($>1200$ ms), and only occasionally at the beginning of viewing time interval when comparing standing to sitting conditions. Some of the observed differences only barely reached the significance level and should therefore be considered as trends.

Our results show that the given task significantly affects both temporal and spatial parameters. Free viewing led to longer fixation durations compared to the guessing task, which replicates previous findings. For example, Mills et al. (2011) also showed that free viewing produces longer fixation durations compared to more specific tasks such as pleasantness rating, memorization, and search tasks. Backhaus et al. (2020) found that guessing tasks resulted in longer fixation durations on average than counting tasks, which proved to be similar to search tasks. Castelhano et al. (2009) could not find significant differences in fixation durations between memorization and search tasks. Therefore, free viewing tasks appear to lead to particularly long fixation durations that cannot be compared to a range of other tasks. It should be noted that caution must be taken when search tasks involve finding people, where we found similarly long fixation durations as in guessing tasks (Backhaus et al. 2020). In summary, the free viewing task might be erroneously perceived as a neutral task, however, experiments show that gaze statistics produced under free viewing are not representative for many other tasks with higher ecological validity.

There are potential effects of repeated viewing in our study. Since we aimed at testing our conditions in a within-subject design, participants in Experiment 1 viewed each image twice. After the free viewing task, the second presentation was investigated with the guessing task (guess the time of the day the image was taken). Consequently, in the resulting task effect of this experiment, it is not possible to disentangle the influence of the task from the influence of repeated viewing, as the images were consistently presented first in the free viewing condition and then in the guessing condition. We consider the fixed (non-randomized) sequence of first the free viewing task and then the guess time task to be inevitable, as in our opinion there is no real alternative to first solving a specific task on a picture and then realizing real free viewing on repeated viewing (i.e. each subject has the option of choosing their own internal task). Rather, the subjects would be primed by the previously specified task.

The effect of repeated viewing of the same image has been investigated before (e.g., Heisz and Shore 2008; Bradley et al. 2011; Kaspar and König 2011; Lancry-Dayan et al. 2019; Trukenbrod et al. 2019). Among others, Kaspar and König (2011) investigated the effects of repeated image viewing for natural scenes and reported, on average, fixation durations that were 2 ms longer for the first repetition (278 ms vs. 280 ms; Kaspar and König, 2011, Fig. 2A), $0.6^\circ$ shorter amplitudes ($5.2^\circ$ vs. $4.6^\circ$; Kaspar and König, 2011, Fig. 2C), and a decrease of 0.06 bit in individual entropy (15.47 bit vs. 15.41 bit; Kaspar and König, 2011, Fig. 2D). The differences we observed in fixation durations during the first 2 s of image viewing clearly exceed the effect of image repetition (250 ms vs. 263 ms). The differences found in saccade amplitudes and entropy fall within the range identified by Kasper and König and could alternatively be explained by the effects of image repetition. It should be noted that the authors listed a variety of other influencing factors, such as motivation or image type, which also have an impact on eye movement parameters.

In Experiment 2, the chosen counting task (count the number of animals shown in the image) results in short fixation durations and a high entropy (Backhaus et al. 2020). Participants rapidly scanned all areas of the images as animals could be located anywhere (on the ground, in the air, in water) and exhibited a wide variety of appearances. The short fixation durations lead to floor effects, while the high entropy values lead to ceiling effects in the data. These effects reduce the possibility of reliably detecting differences between the experimental conditions. In Experiment 2, the analysis of the CFB (Central Fixation Bias) revealed that the distances to the image center, were generally higher compared to Experiment 1, primarily due to the selected task. The strong search behavior in the counting task leads participants to direct their gaze away from the center.

In both experiments, a decrease in entropy during more natural postural positions is observed in combination with a stronger central fixation bias (CFB). One possible interpretation of this finding is that there is a more pronounced anchor effect with mean gaze position close to the image center, which increases CFB and reduces entropy. The only deviation from the observed trend, where the entropy for standing quietly is higher than for sitting without head support in Experiment 2, could potentially be attributed to the fact that sitting on a chair that permits rotations may have induced more movement in the subjects compared to quiet standing. It is important to note that our measurements solely capture gaze position on the image, without distinguishing between eye, head, and trunk movements. These questions would clearly exceed this study’s focus on gaze positions; we did not investigate the physiological production of gaze positions from different body parts, which would require further research with evaluated setups that accurately measure eye, head, and trunk movements independently. Therefore, we do not make any claims regarding the overall movement of the subjects.

Our analyses lend support to the view that body posture and the possibility of movement have a rather limited effect on the spatial eye movement components. However, this should be further investigated with additional conditions, the actual movement of the subjects, and the separate measurement of trunk, head, and eye movements. Our results provide evidence that more natural posture conditions only modestly influence gaze compared to classical laboratory eye-tracking conditions. At the same time, the specific task should be selected with care, both in the laboratory and during more natural experimental settings, since task effects potentially override the more subtle effects of posture.

### Supplementary Information

Below is the link to the electronic supplementary material.

Supplementary file1 (PDF 4300 KB)

## Data Availability

The data and statistical analysis scripts will be made available at https://osf.io/8pc6x/.
