# Supplementary material for: How body postures affect gaze control in scene viewing under specific task conditions
Source: Exp Brain Res. 2024 Feb 1;242(3):745–56. doi: 10.1007/s00221-023-06771-x (PMC11297079; doi:10.1007/s00221-023-06771-x)
Supplement: Supplementary file 1 — Supplementary file1 (PDF 4300 KB) [file 221_2023_6771_MOESM1_ESM.pdf]

Supplementary information

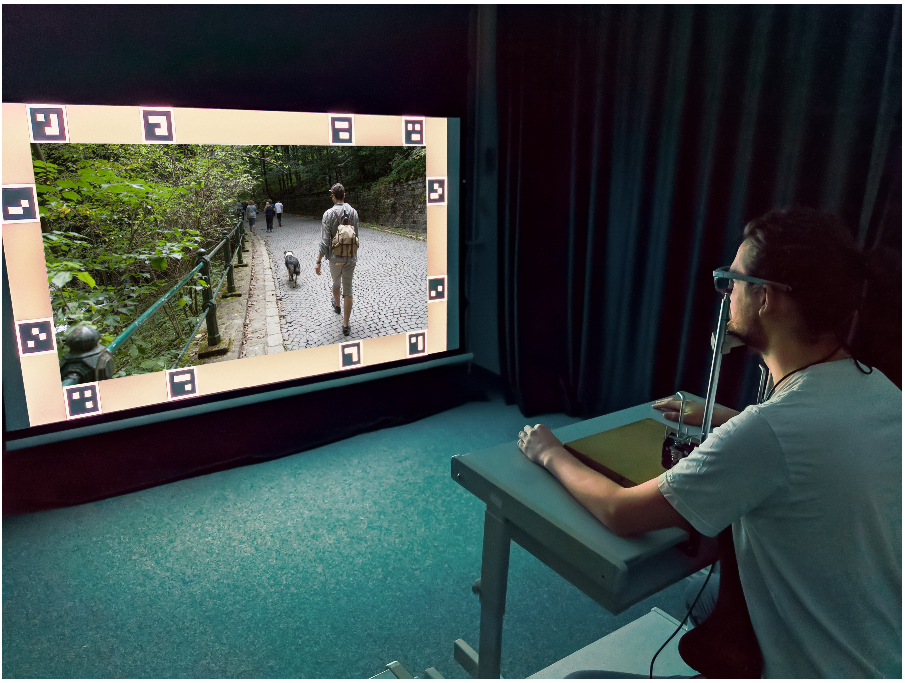

Fig. S1 Laboratory setup: The figure shows the reconstruction of the classical sitting posture with a head support (i.e. a chin rest). Participants are seated 270 cm away from a projection screen whose height is adjusted to the participants' eyes. The subject is equipped with mobile eye-tracking glasses. This setup allows for different body postures (e.g. standing, sitting, turning towards the stimulus material)

Table S1 Experiment 1: LMM – Fixation durations.

	Experiment 1 Log Fixation Duration 0 – 2000 ms			
	β	*SE*	*t*	*p*
Task: Guess_Time - Free_Viewing	-0.03	0.01	**-3.34**	.001
Body: Standing - Chin_Rest	0.00	0.01	0.37	.712
Interaction	0.04	0.02	1.75	.080

Note: Only the fixed effects part is displayed, for detailed information about the random effects refer to the provided code, $|t| > 2$ are interpreted as significant

Table S2 Experiment 1: LMM – Saccade amplitudes.

	Experiment 1<br>Log Saccade Amplitudes 0 – 8000 ms			
	β	*SE*	*t*	*p*
Task: Guess_Time - Free_Viewing	0.05	0.01	**5.18**	<.001
Body: Standing - Chin_Rest	0.02	0.01	1.81	.070
Interaction	-0.00	0.02	-0.05	.964
	Log Saccade Amplitudes 0 – 2000 ms			
	β	*SE*	*t*	*p*
Task: Guess_Time - Free_Viewing	0.05	0.02	**3.00**	.003
Body: Standing - Chin_Rest	0.02	0.02	0.88	.379
Interaction	0.03	0.03	0.74	.46

Note: Only the fixed effects part is displayed, for detailed information about the random effects refer to the provided code, $|t| > 2$ are interpreted as significant

Table S3 Experiment 1: LMM – Entropy.

	Experiment 1<br>Exponentially transformed Entropy			
	β	*SE*	*t*	*p*
Task: Guess_Time - Free_Viewing	-43129.69	15391.02	**-2.80**	.006
Body: Standing - Chin_Rest	-31005.46	10550.38	**-2.94**	.004
Interaction	7607.86	21100.76	0.36	.719

Note: Only the fixed effects part is displayed, for detailed information about the random effects refer to the provided code, $|t| > 2$ are interpreted as significant

Table S4 Experiment 1: LMM – Evolution of the Central Fixation Bias.

	Experiment 1			
	Distance to Image Center[°] 0 – 8000 ms			
	β	*SE*	*t*	*p*
Task: Guess_Time - Free_Viewing	-0.43	0.05	**-8.65**	<.001
Body: Standing - Chin_Rest	-0.28	0.05	**-5.51**	<.001
Interaction	-0.01	0.10	-0.13	.900
	Distance to Image Center[°] 0 – 400 ms			
	β	*SE*	*t*	*p*
Task: Guess_Time - Free_Viewing	-0.09	0.19	-0.50	.617
Body: Standing - Chin_Rest	-0.26	0.19	-1.38	.169
Interaction	0.19	0.37	0.51	.612
	Distance to Image Center[°] 400 - 800 ms			
	β	*SE*	*t*	*p*
Task: Guess_Time - Free_Viewing	-0.40	0.14	**-2.84**	.005
Body: Standing - Chin_Rest	-0.21	0.14	-1.48	.140
Interaction	-0.17	0.28	-0.61	.540
	Distance to Image Center[°] 800 -1200 ms			
	β	*SE*	*t*	*p*
Task: Guess_Time - Free_Viewing	0.17	0.18	0.97	.330
Body: Standing - Chin_Rest	0.09	0.18	0.50	.616
Interaction	-0.29	0.36	-0.80	.421
	Distance to Image Center[°] 1200 - 8000 ms			
	β	*SE*	*t*	*p*
Task: Guess_Time - Free_Viewing	-0.51	0.06	**-9.21**	<.001
Body: Standing - Chin_Rest	-0.31	0.06	**-5.54**	<.001
Interaction	0.01	0.11	0.11	.914

Note: Only the fixed effects part is displayed, for detailed information about the random effects refer to the provided code, $|t| > 2$ are interpreted as significant

Table S5 Experiment 2: LMM – Fixation durations.

	Experiment 2			
	Log Fixation Duration 0 – 2000 ms			
	β	*SE*	*t*	*p*
C1: standing postures - sitting postures	0.01	0.01	0.80	.421
C2: Sitting - Chin_Rest	0.02	0.01	1.85	.064
C3: Balancing - Standing	0.00	0.01	0.18	.858

Note: Only the fixed effects part is displayed, for detailed information about the random effects refer to the provided code, $|t| > 2$ are interpreted as significant

Table S6 Experiment 2: LMM – Saccade amplitudes.

	Experiment 2			
	Log Saccade Amplitudes 0 – 8000 ms			
	β	*SE*	*t*	*p*
C1: standing postures - sitting postures	0.03	0.01	**4.09**	<.001
C2: Sitting - Chin_Rest	0.00	0.01	0.14	.885
C3: Balancing - Standing	0.01	0.01	1.21	.226
	Log Saccade Amplitudes 0 – 2000 ms			
	β	*SE*	*t*	*p*
C1: standing postures - sitting postures	0.04	0.02	**2.92**	.004
C2: Sitting - Chin_Rest	0.03	0.02	1.55	.121
C3: Balancing - Standing	-0.00	0.02	-0.20	.841

Note: Only the fixed effects part is displayed, for detailed information about the random effects refer to the provided code, $|t| > 2$ are interpreted as significant

Table S7 Experiment 2: LMM – Entropy.

	Experiment 2			
	Exponentially transformed Entropy			
	β	*SE*	*t*	*p*
C1: standing postures - sitting postures	-10101.68	8252.99	-1.22	.222
C2: Sitting - Chin_Rest	-23962.39	11671.48	**-2.05**	.041
C3: Balancing - Standing	-31856.52	13684.64	**-2.33**	.021

Note: Only the fixed effects part is displayed, for detailed information about the random effects refer to the provided code, $|t| > 2$ are interpreted as significant

Table S8 Experiment 2: LMM – Evolution of the Central Fixation Bias

	Experiment 2			
	Distance to Image Center[°] 0 – 8000 ms			
	β	*SE*	*t*	*p*
C1: standing postures - sitting postures	-0.15	0.05	**-2.89**	.004
C2: Sitting - Chin_Rest	-0.11	0.07	-1.46	.143
C3: Balancing - Standing	-0.26	0.07	**-3.52**	<.001
	Distance to Image Center[°] 0 – 400 ms			
	β	*SE*	*t*	*p*
C1: standing postures - sitting postures	-0.41	0.16	**-2.47**	.014
C2: Sitting - Chin_Rest	-0.27	0.24	-1.12	.263
C3: Balancing - Standing	0.00	0.23	0.01	.992
	Distance to Image Center[°] 400 - 800 ms			
	β	*SE*	*t*	*p*
C1: standing postures - sitting postures	-0.15	0.14	-1.03	.303
C2: Sitting - Chin_Rest	0.00	0.20	0.02	.981
C3: Balancing - Standing	-0.14	0.20	-0.69	.493
	Distance to Image Center[°] 800 -1200 ms			
	β	*SE*	*t*	*p*
C1: standing postures - sitting postures	-0.12	0.17	-0.67	.501
C2: Sitting - Chin_Rest	0.20	0.25	0.81	.421
C3: Balancing - Standing	-0.27	0.25	-1.07	.285
	Distance to Image Center[°] 1200 - 8000 ms			
	β	*SE*	*t*	*p*
C1: standing postures - sitting postures	-0.13	0.06	**-2.33**	.020
C2: Sitting - Chin_Rest	-0.13	0.08	-1.63	.103
C3: Balancing - Standing	-0.28	0.08	**-3.42**	.001

Note: Only the fixed effects part is displayed, for detailed information about the random effects refer to the provided code, $|t| > 2$ are interpreted as significant
